# Complete genomes of *Clostridium botulinum* type B(F) isolates associated with a 1995 foodborne botulism outbreak from commercial pâté reveals a recombination event disrupting the *ntnh* gene

**DOI:** 10.1099/mgen.0.001169

**Published:** 2024-01-04

**Authors:** Richard A. Harris, Annika Flint, Madeleine Blondin Brosseau, Kelly Weedmark, John W. Austin

**Affiliations:** ^1^​ Bureau of Microbial Hazards, Health Canada, Ottawa, Ontario, Canada

**Keywords:** botulism, botulinum neurotoxin (BoNT), *Clostridium botulinum*, outbreak, foodborne botulism, recombination

## Abstract

Foodborne botulism is a neuroparalytic disease caused by ingestion of foods contaminated with botulinum neurotoxin (BoNT), produced by *Clostridium botulinum*. In 1995 a husband and wife from Québec, Canada, were hospitalized for several months with prolonged muscle paralysis after ingesting a commercial *pâté de campagne*. Examination of faecal samples from both patients and the pâté produced viable Group I (proteolytic) *C. botulinum* type B from each of the three samples. Whole genome sequencing revealed that all three isolates contain identical *bont/B5* and *bont/F2* genes encoded on a plasmid. Both faecal isolate genomes were identical in chromosome and plasmid length, as well as gene content. The genome of the pâté isolate was nearly identical to that of the faecal isolates with the notable difference of a missing 13-gene insertion on the *bont/B5* cluster disrupting the *ntnh* gene. Examination of the insertion revealed several mobile genetic elements that participate in recombination.

## Abbreviations

ANI, average nucleotide identity; BoNT, botulinum neurotoxin; HA, haemagglutinin; NTNH, non-toxic-non-haemagglutinin; T2SS, type II secretion system.

## Data Summary

Data are available under NCBI BioProject PRJNA806856 (GenBank and Sequence Read Archive accessions are listed in Table 2).

### Impact Statement

This study provides the complete genomes of Group I (proteolytic) *Clostridium botulinum* from two faecal isolates (one isolate from each of two patients) and one food isolate from a foodborne botulism outbreak in 1995 involving a commercial pâté. Genome-wide comparisons revealed that the two faecal isolates and the pâté isolate are nearly identical, except for a 13-gene insertion in the *bont/B5* cluster disrupting the *ntnh* gene in both faecal isolates. This suggests that a recent recombination event occurred, either in the pâté, or sometime between ingestion and recovery of the human specimen isolates from culture. Examination of the mobile genetic elements within the insertion may help elucidate the mechanisms contributing to the ongoing diversity of *C. botulinum*.

## Introduction

Botulism is a neuroparalytic disease caused by exposure to botulinum neurotoxin (BoNT) produced by the Gram-positive spore-forming obligate anaerobic bacterium *Clostridium botulinum*. BoNTs function by inhibiting acetylcholine release from presynaptic neurons at neuromuscular junctions leading to widespread and prolonged muscle paralysis. Foodborne botulism is caused by contamination of foods by *C. botulinum* and subsequent growth and production of BoNT that is ingested. As *C. botulinum* is ubiquitous in the environment [1], food safety considerations for botulism are focused on destruction of the organism through high temperature or combined high pressure/high temperature treatments, as well as prevention of growth by storage at low pH, low water activity, and refrigerated or freezing temperatures [2].

There are six main species of BoNT-producing clostridia, categorized based on physiological properties, and seven serotypes of BoNT (designated alphabetically A–G) based on neutralization of toxicity by serotype-specific antisera. *C. botulinum* Group I (proteolytic) and Group II (non-proteolytic) are most commonly associated with human illness and produce BoNT types A, B, F, and B, E, F, respectively [3]. Subtypes of BoNT can differ from each other in amino acid sequence from 7 % (*bont/B*) to 36 % (*bont/F*) and can be located on a plasmid or chromosome [4]. BoNTs form complexes with accessory proteins haemagglutinin (HA) and non-toxic-non-haemagglutinin (NTNH) to facilitate absorption across the gastrointestinal tract [5]. Genes encoding the BoNTs and accessory proteins, along with transcriptional regulators, are arranged in a *bont* gene cluster at a limited number of specific sites on the chromosome and/or a plasmid [6].

Whole genome sequencing revealed that Group I *C. botulinum* has a relatively stable genome except for the *bont* gene cluster [7–9]. Most of these clusters are flanked by insertion sequence elements encoding transposases that may be capable of horizontal gene transfer between *C. botulinum* Groups I and II, or between species such as the insertion of *bont/F7* into *Clostridium baratii* [10–12]. These insertion sequence elements were found to be degraded from their full-length elements, indicating a remnant of the evolutionary history of *C. botulinum* rather than currently active mobile elements [13]. There are several descriptions of chimeric and mosaic *ntnh* genes from Group I *C. botulinum* isolates, which indicates this is a ‘hot spot’ for recombination events [9, 14–16]. In one of these events, a mosaic *ntnh* gene was discovered with the first half that was 97 % identical to those from *bont/C* strains and the second half 93 % identical to those of *bont/A2* strains [15]. Another recombination event described a *bont/A1* gene insertion into the *ntnh* gene within an *ha* cluster, which is usually associated with *bont/B* strains [14]. Little is known about how mobile elements currently function to facilitate recombination events in *C. botulinum*.

In 1995, a foodborne botulism outbreak from a commercial *pâté de campagne* caused a husband and wife to become hospitalized. Isolates of viable Group I *C. botulinum* were obtained from both faecal specimens and the consumed pâté. Genome-wide comparisons of all three *C. botulinum* isolates confirmed a high degree of homology and high conservation of gene synteny. The plasmids from both faecal isolates contained an 11 kb insertion on the *bont* cluster disrupting the *ntnh* gene, yet the isolate from the pâté did not contain the insertion. This suggests that an insertion or deletion recombination event occurred either in the pâté or before isolate recovery (after ingestion or during culture).

## Methods

### Case description

In 1995, a husband and wife from Québec, Canada, were admitted to hospital a week after ingesting a commercial *pâté de campagne*. The wife displayed vision problems, respiratory distress and dysarthria. She was originally diagnosed with a cerebro-vascular incident, but the following day the husband displayed respiratory distress and dysarthria, at which point the diagnosis changed to suspected foodborne botulism. Both patients received antitoxin and were intubated. After a month in hospital, the husband still needed help to move his limbs and the wife was still unable to move her legs. All clinical and food specimens were tested for BoNT and viable *C. botulinum* by the Health Canada Botulism Reference Service for Canada laboratory in Ottawa, Ontario, using the mouse bioassay according to MFHPB-16 [17]. Serum, gastric contents and faeces tested negative for BoNT, but both patients’ faeces were positive for viable Group I *C. botulinum* type B. The remaining commercial pâté also tested negative for BoNT but positive for viable Group I *C. botulinum* type B. The ingredients of the pâté were as follows: ground veal, water, wheat crumbs, onions, salt, chopped garlic and spices.

### Bacterial isolates and growth conditions

Faecal samples were diluted approximately 3 ml g^−1^ with gelatin phosphate buffer (0.2 % gelatin and 0.4 % Na_2_HPO_4_) and homogenized by vortexing. Extracts of the pâté were prepared by mixing 65 g with 44 ml of gelatin phosphate buffer and homogenizing in a Seward 400 Circulation Stomacher. Enrichments of the faeces and pâté were performed by inoculating 25 ml of TPGY (5 % tryptone, 0.5 % peptone, 0.4 % glucose, 2 % yeast extract and 0.1 % sodium thioglycolate) and incubating at 35 °C for 24 h and then 30 °C for 4 days under anaerobic conditions. Cultures were streaked onto *Clostridium botulinum* isolation (CBI) agar [18] and incubated at 30 °C for 4 days. Single-colony picks of PA9508B (pâté consumed by both patients), FE9508BRB (faecal from patient 1) and FE9508BPD (faecal from patient 2) were grown on CBI agar for 48 h at 35 °C under anaerobic conditions and frozen at −80 °C using Microbank cryovials (Pro-Lab Diagnostics) without further subculture. In 2022, frozen stock was streaked onto McClung Toabe Egg Yolk Yeast Extract (MT-EYE) agar [1.5 % McClung-Toabe agar (Difco), 5 % egg yolk extract and 5 % yeast extract (Difco)] and incubated overnight at 35 °C anaerobically, then cells from a single-colony 25 ml TPGY inoculate were collected after 24 h of anaerobic growth at 35 °C.

### DNA extraction and sequencing

Cell pellets were resuspended in 1 ml of gelatin phosphate buffer and 1 ml of 2× Zymo DNA/RNA Shield (Cedarlane) and held at 4 °C. DNA extractions were performed using the Zymo Quick-DNA HMW MagBead kit (Cedarlane) according to the manufacturer’s protocols (RNAse A treatment without enzymatic lysis). Illumina libraries were constructed using the NexteraXT DNA Library Preparation Kit and paired-end sequencing was performed on a MiSeq instrument (v3 chemistry, 2×300 bp) according to the manufacturer’s instructions (Illumina). For Oxford Nanopore sequencing, DNA was first concentrated to ≥50 ng ml^−1^ in 10 mM Tris/Cl, pH 8, using 0.8 volumes of AMPureXP beads (Beckman Coulter) and prepared with the rapid barcoding sequencing kit (SQK-RBK004) for 1D MinION sequencing (R9.4 FLO-MIN106 flow cell) as per the manufacturer’s instructions (Oxford Nanopore Technologies).

### Genome assembly and annotation

Raw Illumina reads were processed using FastP v0.20.1 to remove adapter and barcode sequences, correct mismatched bases in overlaps, and filter low-quality reads (Q<20) [19]. Long-read signal processing, base calling, demultiplexing and adapter trimming were performed using Guppy GPU v5.0.16+b9fcd7b (https://community.nanoporetech.com/downloads), and reads of <1 kb were removed using Filtlong v0.2.0 (github.com/rrwick/Filtlong). Filtered reads were assembled *de novo* with Unicycler v0.4.8 in normal mode [20] for PA9508B and FE9508BPD, and with Trycycler v0.3.3 (cluster, reconcile, partition and consensus functions; default circularization and rotation) [21], Flye v2.8.1-b1676 [22], Raven v1.2.2 (github.com/lbcb-sci/raven), Canu v2.2 [23] and Necat v0.0.1 [24] for FE9508BRB. Error correction was performed with Medaka v1.1.3 (github.com/nanoporetech/medaka), Polypolish v0.4.3 [25] and Polca v4.0.5 (github.com/alekseyzimin/masurca/releases). Assemblies were annotated with PGAP v2022-04-14.build 6021 (best-placed reference protein set, GeneMarkS-2+) (github.com/ncbi/pgap) and analysed with QUAST v5.0.2 (github.com/ablab/quast).

### Genome analysis and *in silico* typing

Average nucleotide identity (ANI) between *C. botulinum* genomes was calculated using FastANI v1.33 [26], using PA9508B, FE9508BRB or ASM17107 (GCA_000171075.1) as references. *C. botulinum* genomes were aligned using Minimap2 v2.24-r1122 with the -x asm5 parameter [27] and processed using paftv v0.1.0 using the -X option (github.com/MoinSebi/paftv). Alignments were visualized with AliTV v1.0.6 [28] using a minimum link identity and length of 90 % and 10k, respectively [29] and Adobe Illustrator CS5. Proksee (proksee.ca) and Adobe Illustrator CS5 were used to visualize chromosome and plasmid sequences. PHASTEST (phastest.ca) and DBSAN-SWA (github.com/HIT-ImmunologyLab/DBSCAN-SWA) were used to identify prophage-like regions. mega v10.1.8 [24] was used to align BoNT and OrfX/HA sequences using a subset of reference sequences from BoNTbase (bontbase.org) (github.com/bmh-genomics/bont_aa-typing, f9caf33) and from Uniprot (uniprot.org) (github.com/bmh-genomics/orfx-ha_cluster_DB, 6e13cb2), respectively. Maximum-likelihood phylogenetic trees based on the Jones–Taylor–Thornton model were reconstructed using 1000 bootstrap replicates. Percentage nucleotide identities for the *bont/B5* and *bont/F2* genes were determined by blast analysis (blast.ncbi.nlm.nih.gov/Blast.cgi) using reference sequences from github (github.com/bmh-genomics/bont_aa-typing) and GenBank (ncbi.nlm.nih.gov/genbank). Mutation prediction was performed using the Breseq v0.35.5 pipeline and default parameters [30]. Variants were identified using the Illumina reads for each isolate against either the PA9508B or FE9508BRB closed genome as a reference. Illumina and Nanopore reads were mapped using BBMAP v38.18 using default parameters (sourceforge.net/projects/bbmap) and Minimap2 v2.24-r1122 using the -ax map-ont parameter [24], respectively. Coverage plots were made using Artemis v18.2.0 [31].

## Results

**Fig. 1.**
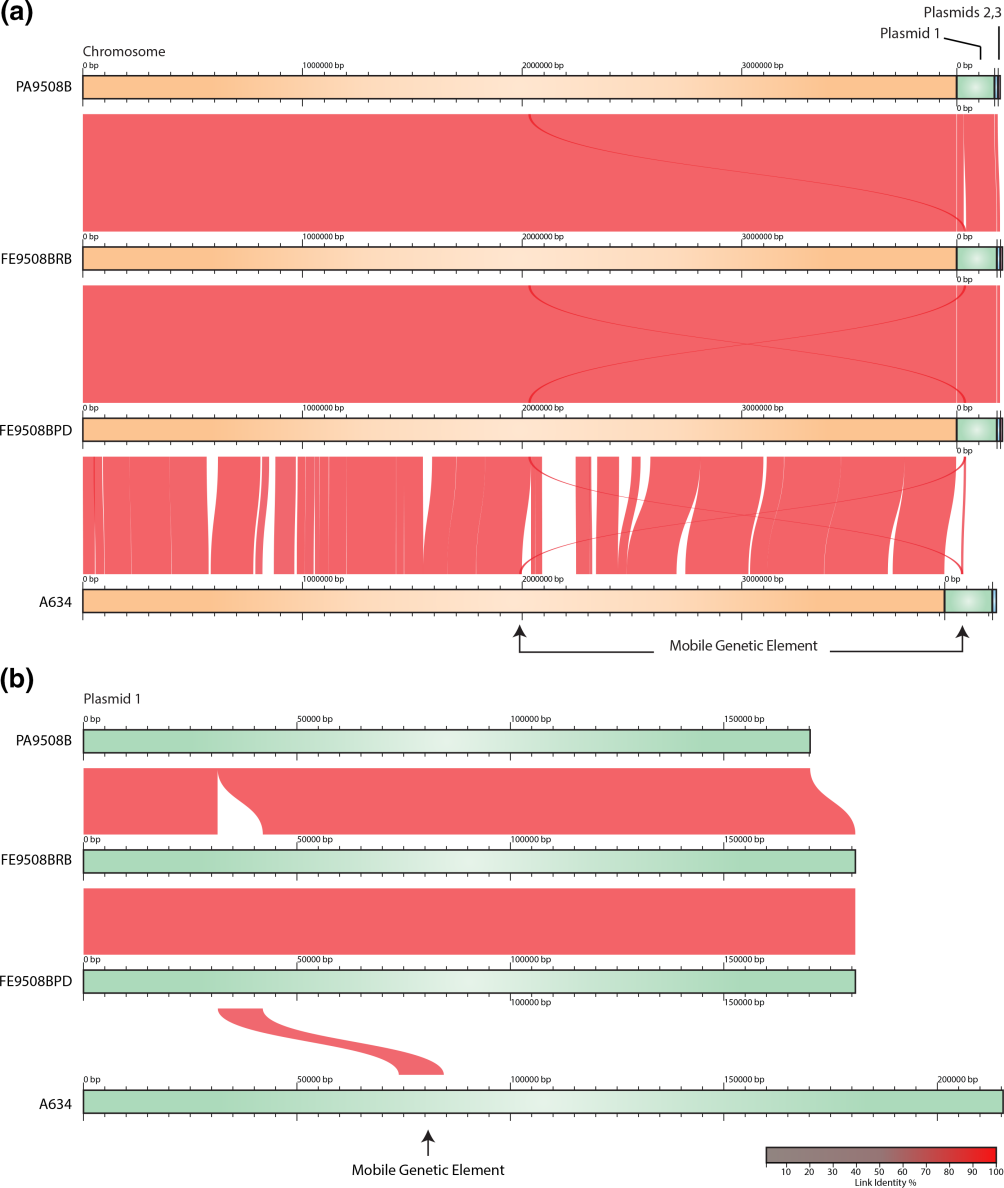
*C. botulinum* pâté (PA9508B) and faecal (FE9508BRB, FE9508BPD) isolates are highly homologous and show conserved gene synteny. (**a**) Multiple genome alignment of *C. botulinum* isolates. Duplication of an incomplete putative prophage is observed in FE9508BRB, FE9508BPD and A634. (**b**) Multiple alignment of plasmid sequences showing location and identity of a mobile genetic element. Linked segments represent orthologous gene pairs between genomes and breaks in synteny are shown as blank spaces. Colour denotes the percentage similarity of links between isolates.

*C. botulinum* genome sequencing, assembly and annotation metrics are shown in Tables 1 and 2. All three isolates contain a chromosome and three plasmids. Genome-wide comparisons show high homology between isolates across the length of the chromosome and high conservation of gene synteny (Fig. 1a). The faecal isolates, FE9508BRB and FE9508BPD, were 100 % identical by ANI with each having the same chromosome length (3 977 274 bp) and plasmid lengths (180 817, 13 761 and 9 082 bp, respectively). Isolates FE9508BRB and FE9508BPD also contained the same gene content (3839 genes, 24 rRNAs, 81 tRNAs and one CRISPR/Cas system). The pâté isolate, PA9508B, was nearly identical to the faecal isolates with respect to ANI score (99.99 %), chromosome length (3 977 270 bp), plasmid lengths (170 213, 13 761 and 9 082 bp, respectively) and content (3826 genes, 24 rRNAs, 81 tRNAs and one CRISPR/Cas system). PA9508B, FE9508BRB and FE9508BPD are also highly similar (99.97 %) by ANI score to the *C. botulinum* Bf strain ASM1707 (GCA_000171075.1).

**Table 1.** Sequencing summary

Isolate	Sequencing technology	Raw reads (no.)	Average raw length (bp)	Raw fold coverage	Filtered reads (no.)	Average filtered length (bp)
PA9508B	Illumina	1 748 312	175	73.5	1 710 918	174
	ONT	413 257	2316	229.6	413 257	2316
FE9508BR	Illumina	2 123 690	175	89.3	2 076 774	175
	ONT	405 360	1809	175.4	192 295	3288
FE9508BP	Illumina	1 613 204	182	70.5	1 577 700	181
	ONT	413 078	1564	154.6	194 026	2788

**Table 2.** Isolate provenance, NCBI accession numbers, assembly and PGAP annotation metrics of *C. botulinum* genomes studied (BioProject PRJNA806856)

Metric	PA9508B	FE9508BRB	FE9508BPD
Sample	Food (pâté)	Clinical (faeces)	Clinical (faeces)
BoNT typing	B5 (silent F2)	B5 (silent F2)	B5 (silent F2)
Bioproject	PRJNA806856	PRJNA806856	PRJNA806856
GenBank	CP102917–CP102920	CP102913–CP102916	CP102909–CP102912
Illumina SRA	SRR18052219	SRR18052218	SRR18052217
Nanopore SRA	SRR18052216	SRR18052215	SRR18052214
Chromosome (bp)	3 977 270	3 977 274	3 977 274
Plasmid 1 (bp)	170 213	180 817	180 817
Plasmid 2 (bp)	13 761	13 761	13 761
Plasmid 3 (bp)	9082	9082	9082
GC content (%)	28.06	28.05	28.05
Annotated features	4014	4029	4029
Genes	3826	3839	3839
rRNAs	24	24	24
tRNAs	81	81	81
Non-coding RNAs	4	4	4
Pseudogenes	79	81	81
CRISPR/Cas system	1	1	1

The PA9508B, FE9508BRB and FE9508BPD genomes each contain full-length *bont/B5* and *bont/F2* genes [100 % nucleotide identity with CDC4013 *bont/B5* and CDC3281 *bont/F2*, respectively (Fig. S1, available in the online version of this article)] encoded on plasmid 1. For each isolate, the *bont/B5* gene is located within an *ha+orf–* cluster and the *bont/F2* gene is in an *ha–orf+* cluster. Interestingly, the mouse bioassay revealed that each of these isolates are type BoNT/B (silent F).

**Fig. 2.**
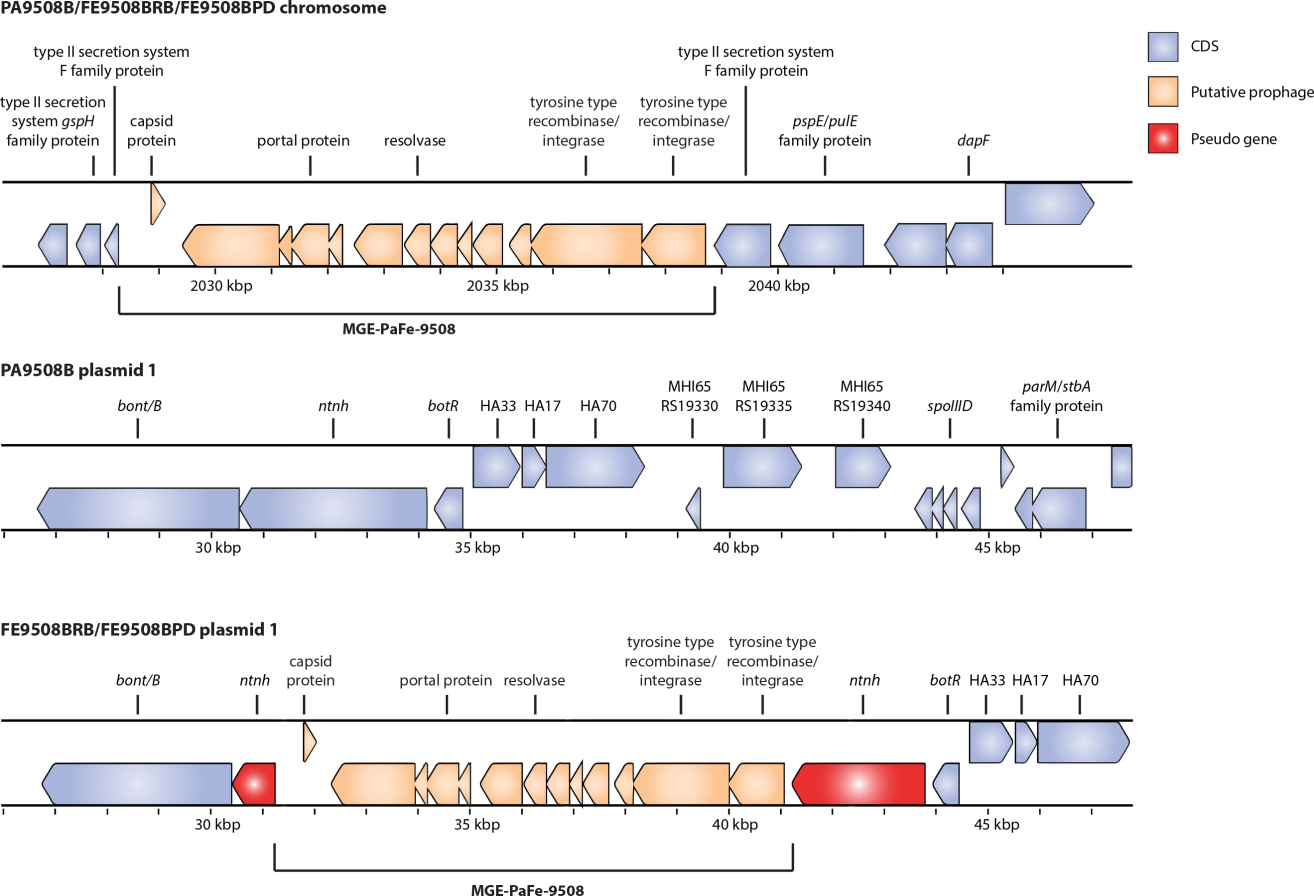
Genome comparison of the *bont/B* cluster on plasmid 1 of *C. botulinum* pâté PA9508B and faecal FE9508BRB and FE9508BPD isolates. FE9508BRB and FE9508BPD contain a 10.6 kb insertion of 13 genes disrupting *ntnh* on plasmid 1. A nearly identical 10.6 kb sequence (99 %) is also found on the chromosome in all three isolates within a type II secretion system (T2SS) gene cluster. MGE-PaFe-9508 refers to the 10.6 kb region on the chromosome and plasmid of these isolates.

**Fig. 3.**
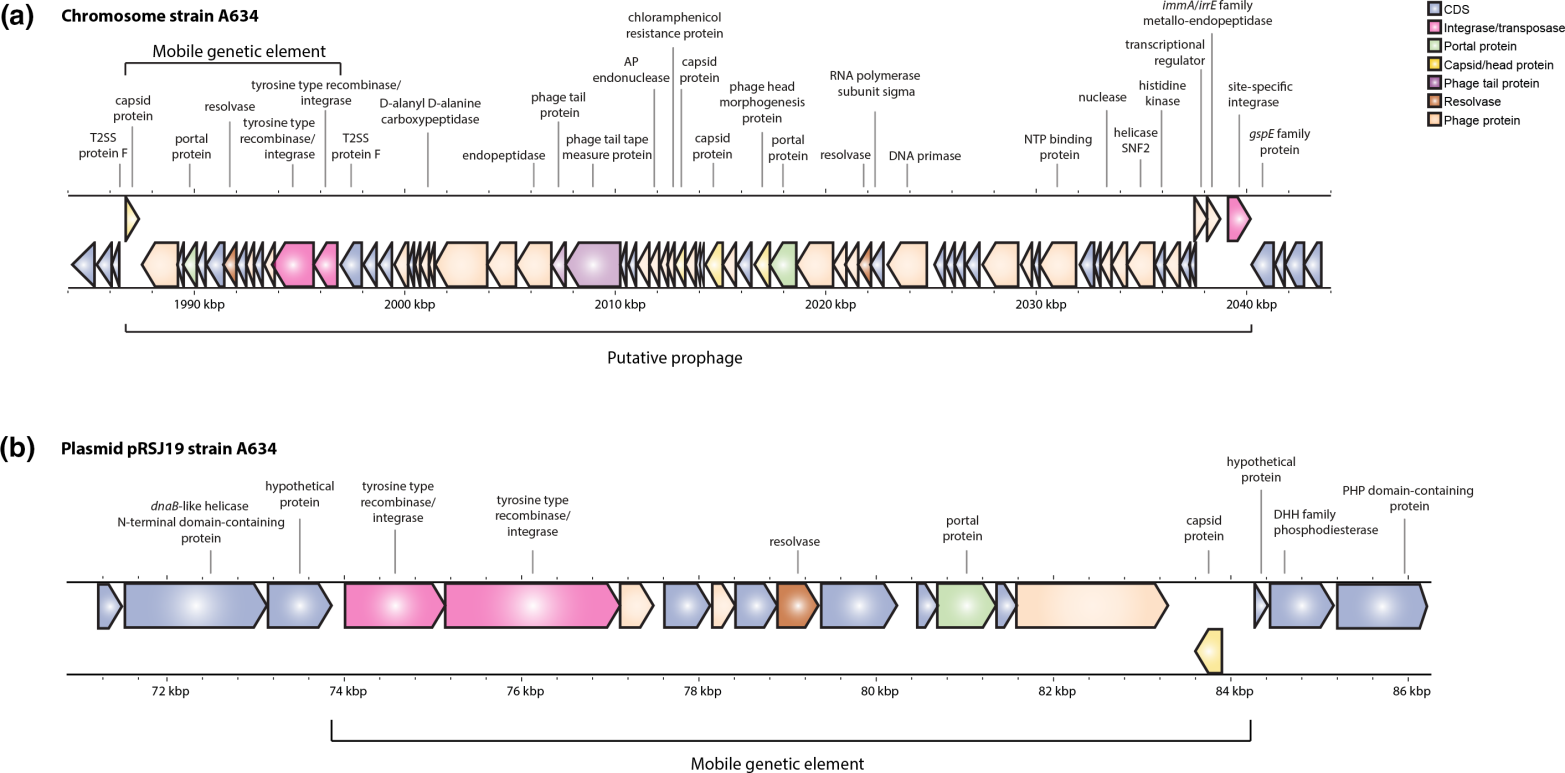
*C. botulinum* strain A634 putative prophage. (**a**) Chromosomal location and gene content of the putative intact prophage region (~54 kb) within a type II secretion system (T2SS) gene cluster. A 10.6 kb, incomplete mobile element, 98 % identical to MGE-PaFe-9508, is located at the 5′ end of the putative prophage. (**b**) Plasmid pRSJ19 showing the location and gene content of the incomplete putative prophage.

A major difference in gene synteny was observed in the faecal isolates (FE9508BRB and FE9508BPD), which both contain an 11 kb insertion on plasmid 1 disrupting the *ntnh* gene of the *ha+orf–* (*bont/B5*) cluster, compared to PA9508B (Fig. 2). The insertion (hereafter named MGE-PaFe-9508) contains 13 intact genes, including: a capsid protein, DUF2326 domain-containing protein, ABC three-component system middle component 6 protein, portal protein, P63C domain-containing protein, resolvase, rRNA methyltransferase, DUF6262 family protein, three hypothetical proteins and two tyrosine-type recombinase/integrases. blast analysis of the protein sequences found that the DUF2326 domain-containing protein (MHI66_RS19310, MHB86_RS19310) also contains an Smc domain and shares 43 % identity with a chromrosome segregation ATPase in *Caudoviricetes* sp. (GenBank accession DAM13490). Although the precise function of the Smc domain in this protein is not known, Smc domains function in DNA binding and segregation [32]. A nearly identical (99 %) 11 kb region was also found on the bacterial chromosome in all three isolates (Fig. 2) within a cluster of genes encoding a type II secretion system (T2SS) (coordinates: ~2028–2038 kb). Based on the presence of several prophage genes (i.e. capsid protein, portal protein, resolvase, two tyrosine-type recombinase/integrases), prophage prediction software was used to determine if MGE-PaFe-9508 encodes a putative prophage. Neither PHASTEST nor DBSCAN-SWA identified the region as a prophage on the chromosome (PA9508B, FE9508BRB, FE9508BPD) or on plasmid 1 (FE9508BRB, FE9508BPD). Of note, the default settings of these tools require a minimum of six phage-like genes within a maximum gene distance of 3000 bp, or within a sliding window of 60 proteins, to identify a region as a prophage. MGE-PaFe-9508 only contains five phage-like genes and was therefore identified as an incomplete prophage.

blast analysis of MGE-PaFe-9508 revealed 98 % sequence identity to a genomic region in *C. botulinum* type A4 strain A634 (CP013845.1) on the bacterial chromosome as well as its plasmid pRSJ19_2 (CP013844.1) (data not shown). Similar to the *C. botulinum* isolates in this study, the genomic region is located within a T2SS on the chromosome. However, other than the 13-gene region, there is no significant gene synteny between plasmid 1 of FE9508BRB or FE9508BPD and pRSJ19_2 of strain A634 (Fig. 1b). Inspection of the *C. botulinum* A634 strain revealed the presence of additional prophage-related genes downstream from the tyrosine-type recombinase/integrases on the chromosome (Fig. 3a). Prophage prediction of the A634 genome identified this region as a putative prophage. PHASTEST identified a prophage region of 67.7 kb from 1 990 548 to 2 052 276 bp (which excludes the capsid gene) and DBSCAN-SWA of 59.3 kb from 1 986 946 to 2 046 337 bp (includes the capsid gene). Furthermore, although the prophage identification tools predict a single complete prophage ranging from ~59 to 68 kb, it is also possible that this region codes for two prophages: the incomplete 13-gene region also observed in the pâté outbreak isolates, and an adjacent complete prophage downstream.

Analysis of pRSJ19_2 from *C. botulinum* A634 shows that only the putative incomplete prophage (MGE-PaFe-9508 on plasmid 1 of FE9508BRB and FE9508BPD) is encoded (Fig. 3b). Since the remaining genes of the chromosomal prophage (Fig. 3a) are absent (Fig. 3b), this observation suggests that MGE-PaFe-9508 is a mobile genetic element capable of movement to other genomic locations. Furthermore, the presence of the region on a plasmid also suggests potential for horizontal transfer to other bacterial strains.

To investigate the possibility of MGE-PaFe-9508 being an active mobile element in PA9508B, FE9508BRB and FE9508BPD, read coverage was mapped across the insert region on the chromosome and plasmid using Nanopore long-read data (Fig. S2). Long-read data were used to ensure unique and specific mapping of the reads to either the chromosome or plasmid (specific mapping was not possible using short-read Illumina data). The chromosome of PA9508B showed no significant increase in coverage across MGE-PaFe-9508 (Fig. S2a). Interestingly, an increase in chromosomal (~2.5×) and plasmid (~2×) read coverage was observed in both FE9508BRB and FE9508BPD spanning MGE-PaFe-9508 (Fig. S2a,b). This result suggests this mobile genetic element may be active in FE9508BRB/FE9508BPD, but not in PA9508B.

**Fig. 4.**
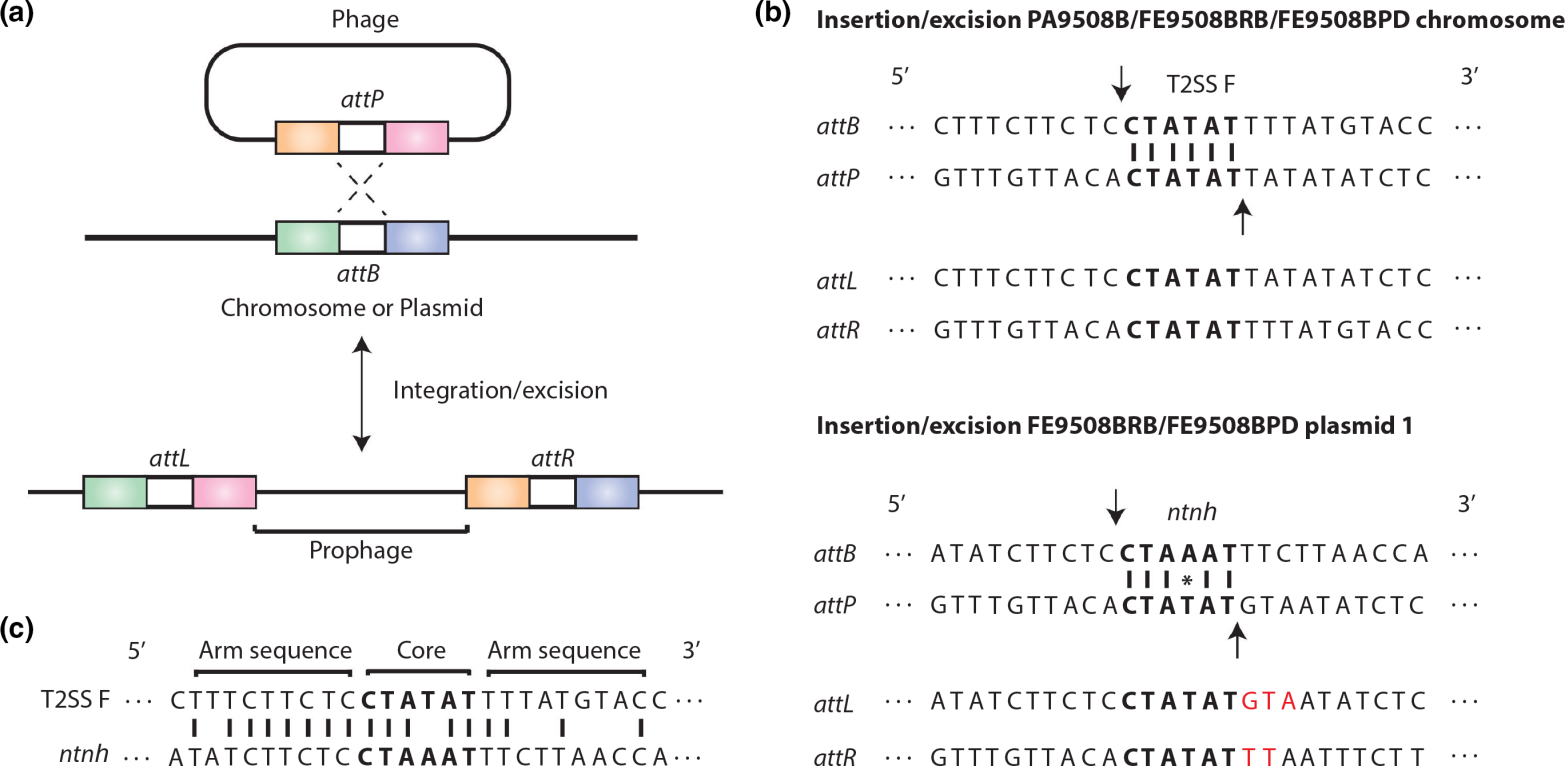
Putative chromosomal and plasmid attachment sites of the 13-gene insertion sequence, MGE-PaFe-9508, of *C. botulinum* FE9508BRB and FE9508BPD. (**a**) Schematic of the insertion mechanism of phage regions into specific chromosome or plasmid locations via attachment sites *attP* and *attB*, respectively. (**b**) Alignment of attachment sequences from type II secretion system (T2SS) F family protein or *ntnh* (*attB*), phage (*attP*) and the MGE-PaFe-9508 incomplete putative prophage (*attL* and *attR*). Core sequences are in bold, cleavage sites are indicated by arrows and differences between attachment sequences are shown in red. (**c**) Alignment of chromosome and plasmid regions at the phage integration site. Core and arm sequences are shown as well as identical base matches between the T2SS F family protein and *ntnh*.

As MGE-PaFe-9508 appears to be active based on the sequencing coverage analysis, the *ntnh* pseudo gene on plasmid 1 of FE9508BRB and FE9508BPD was analysed to determine potential binding sites (Fig. 4). A precise insertion location can be clearly defined in the disrupted *ntnh* gene. Thus, the putative attachment sites for the tyrosine-type recombinase/integrase (*attL* and *attR*) were identified and the overlapping sequences were used to determine *attB* and *attP* (Fig. 4b). The *attB* site was determined to most likely be CTAAAT, whereas *attP* was CTATAT. Interestingly, the *attB* and *attP* sites have a mismatched base at the binding site. Similarly, the *attB*, *attP*, *attL* and *attR* sites were identified for the MGE-PaFe-9508 insert region on the chromosome of PA9508B, FE9508BRB and FE9508BPD (Fig. 4b). The *attB* and *attP* sites were both determined to be CTATAT. These *attP* sites are consistent with a recombination event involving the mismatched base at the core site on plasmid 1. There is a three-base difference of GTA versus TAT on the arm sequences of the predicted phage sequences as well as a two-base difference in the *attR* arm sequence on the plasmids (Fig. 4b). The origin of these differences is unclear but could have arisen during the recombination process or by mutation. Furthermore, we noted 8 bp of sequence identity of *ntnh* at the arm sequence with the chromosomal gene T2SS protein F (which is directly upstream of the chromosomal MGE-PaFe-9508 insertion site) (Fig. 4c).

Single nucleotide variant analysis based on Illumina read mapping did not identify any variants between the faecal and food isolates; however, both the faecal isolates contain an extra series of nucleotides (CTCAA) in an intergenic region between the 16 and 23S rRNA genes compared to the PA9508B isolate (genes MHI65-000059/MHI65-000060, 57 506 bp in PA9508B) (data not shown).

## Discussion

All three isolates contain an identical full-length *bont/B5* gene located in an *ha+orf*− cluster and a full-length *bont/F2* gene located in an *ha–orf+* cluster encoded on plasmid 1. Two types of *bont* clusters are typically found in *C. botulinum*: HA gene clusters that are associated with BoNT types A1, B, C, D and G; and ORF clusters that are associated with BoNT types A2, E and F [33]. Both HA and ORF clusters contain the *ntnh* gene. Considerable variability exists between *bont* clusters for different Group I *C. botulinum* strains in terms of accessory gene content, genomic arrangement and sequence homology [34]. Yet, *bont* clusters have genes that are highly conserved and can show a significant degree (95–100 %) of sequence identity over large segments [9, 35]. Indeed, the *bont/B5* and *bont/F2* genes from all three isolates share 100 % nucleotide identity with CDC4013 *bont/B5* and CDC3281 *bont/F2*, respectively. All three isolates were found to produce BoNT/B (silent F) by mouse bioassays. Most strains of *C. botulinum* produce only a single toxin type, yet dual-toxin strains of type Bf have been previously characterized that produce both B (predominantly) and F [36–38]. To our knowledge the only previous report of *C. botulinum* of type B(F) that harbours a silent *bont/F* gene was isolated from a 1989 botulism outbreak in yoghurt in the UK that was originally typed as BoNT/B and subsequently sequenced as type B5/F2 [34, 39].

The two faecal isolates contained an 11 kb insertion, termed MGE-PaFe-9508, disrupting the *ntnh* gene of the plasmid *ha+orf*− (*bont/B5*) cluster, yet the pâté isolate did not. Because the faecal and food isolates are otherwise clonal, this suggests that a recent recombination event occurred that resulted in an 11 kb insertion into the *ntnh* gene, or a deletion of the 11 kb region from the *ntnh* gene. Although a precise mechanism for this recombination event has not been experimentally demonstrated, sequencing and bioinformatic analysis can be used for insight. A nearly identical segment was found on the chromosome in all three isolates within a cluster of genes encoding a T2SS, suggesting that this was the origin of the insertion. The *ntnh* gene is a known ‘hot spot’ for recombination, and examples of *C. botulinum* strains containing mosaic *ntnh* genes have been previously described [9, 14–16]. The 13-gene insertion in the faecal isolates probably encodes an active, incomplete putative prophage, MGE-PaFe-9508, which is able excise itself from the genome and replicate, but lacks canonical holin and endolysin genes to induce lysis. The insert encodes several intact genes that may play a role in transposition. This includes the transposon-associated resolvase protein that is involved in recombination and insertion events in the *bont* gene cluster of *C. botulinum* [9]. MGE-PaFe-9508 also has two tyrosine-type recombinases/integrases that are a large family of proteins that function in the transposition of mobile genetic elements in prokaryotes [40]. The increased sequence coverage spanning MGE-PaFe-9508 in the faecal isolates further supports an active prophage leading to the recombination event in the *ntnh* gene. The presence of this 13-gene insertion in other *C. botulinum* strains (i.e. A634), both on the chromosome and on the plasmid, also strongly suggests MGE-PaFe-9508 is active and capable of transposition. Additionally, we identified putative binding (*att*) sites in the *ntnh* gene that may be involved in the integration and/or excision of this insert sequence, and sequence homology between *ntnh* and the T2SS family protein at the site of integration suggests this is a targeted region for recombination.

Examination of strain A634 by prophage identification software detected an intact prophage region (67.7 kb by PHASTEST) containing a highly similar sub-region (98 % identity) to MGE-PaFe-9508 in our isolates; yet, this complete prophage is absent in our isolates. It remains unknown if this intact prophage was originally present on the chromosome of PA9508B, FE9508BRB and FE9508BPD, and subsequently lost or degraded, or if the isolates only ever encoded the smaller, incomplete prophage. It also remains unclear why the intact putative prophage of A634 is not present on plasmid pRSJ19. This observation could potentially be explained if A634 encodes two adjacent prophages (i.e. the incomplete MGE-PaFe-9508 prophage and a smaller complete prophage). Interestingly, PHASTEST analysis identified four intact prophages (size range ~24–73.7 kb) on the chromosome of all faecal and food isolates studied (Fig. S3). Thus, it is tempting to speculate that MGE-PaFe-9508 observed in FE9508BRB and FE9508BPD on plasmid 1 may be a satellite phage. Satellite phages are small phages that rely on helper bacteriophages for propagation, can encode widespread functions (i.e. antibiotic resistance, virulence, defence) and transfer horizontally between bacteria [41]. The presence of intact chromosomal prophages on PA9508B, FE9508BRB and FE9508BPD (Fig. S3) could provide the replicative and structural factors required for satellite phage function.

The NTNH protein is an accessory to BoNTs along with a combination of HA proteins that together comprise a high-molecular-weight progenitor toxin complex in various different forms that are essential to oral toxicity [5]. NTNH plays a specific role in binding directly to BoNTs and shielding them from the low pH environment of the gastrointestinal tract [42], while HA proteins interact with intestinal epithelial cells and facilitate absorption into the circulatory system [43]. Considering that the MGE-PaFe-9508 insertion disrupted the *ntnh* gene in the faecal isolates, it is possible this affected pathogenicity for these isolates through altered pathogen/host interactions. However, the mouse bioassay did not determine a difference in pathogenicity between the pâté and faecal isolates, possibly due to the fact that intraperitoneal injection bypasses the gastrointestinal tract altogether.

A blast alignment of the insertion (using databases accessed on 15 March 2023: GenBank, EMBL, DDBJ, PDB, RefSeq) did not identify any other *C. botulinum* genomes that contain the same insert within the *ntnh* gene. Furthermore, blast alignments to other Group I *C. botulinum* type B5/F2 genomes from previous outbreaks [36, 39, 44, 45] did not contain disruption of the *ntnh* gene(s). This indicates that the insertion within the *ntnh* gene is a unique feature of these faecal isolates, and further supports the notion that this is an example of a recombination event.

In sum, these results describe a 1995 outbreak of foodborne botulism acquired from a commercial *pâté de campagne* affecting a husband and wife. The two faecal isolates and one pâté isolate of Group I *C. botulinum* type B5(F2) were nearly identical with the key difference of an insertion disrupting the *ntnh* gene within the *bont* cluster of both faecal isolates, but not the pâté isolate. This is likely to be the result of an insertion or deletion recombination event occurring either in the pâté, or before isolate recovery (after ingestion or during culture). Further examination of these associated mobile genetic elements may help elucidate the mechanisms that mediate the ongoing genetic diversity of *C. botulinum*.

## Supplementary Data

Supplementary material 1
