# Supplementary material for: Complete genomes of Clostridium botulinum type B(F) isolates associated with a 1995 foodborne botulism outbreak from commercial pâté reveals a recombination event disrupting the ntnh gene
Source: Microb Genom. 2024 Jan 4;10(1):001169. doi: 10.1099/mgen.0.001169 (PMC10868621; doi:10.1099/mgen.0.001169)
Supplement: Supplementary material 1 [file mgen-10-1169-s001.pdf]

Supplementary Material – Figure Legends

Supplementary Figure 1. Phylogenetic analysis of *C. botulinum* neurotoxin proteins. Multi-protein alignments were generated using MUSCLE and Maximum Likelihood tree constructed using MEGA (v110.1.8) with 1000 bootstrap replicates. The scale bar represents the phylogenetic distance expressed as amino acid substitutions per site. Bootstrap values are shown at each node. Reference sequences were obtained from BoNTbase (bontbase.org) with serotypes, subtypes, strains, NCBI accession and UniprotKB accession numbers shown.

Supplementary Figure 2. Increased coverage depth at insertion sequence is observed in *C. botulinum* fecal isolates FE9508BRB and FE9508BPD but not pâté isolate PA9508B. Nanopore long-reads were mapped to respective *C. botulinum* genomes using MiniMap2 and visualized using Artemis. (A) Heat maps and coverage depth of the insert and flanking regions on the chromosome of PA9508B, FE9508BRB and FE9508BPD. (B) Heat maps and coverage depth of the insert and flanking regions on plasmid 1 of FE9508BRB and FE9508BPD. Green; PA9508B; Blue: FE9508BRB; Black: FE9508BPD.

Supplementary Figure 3. PA9508B, FE9508BRB, and FE9508BPD encode four intact putative prophages on the chromosome. Intact prophage regions of 42.5 kb, 24.2 kb, 73.7 kb and 50.4 kb are shown in green, A questionable prophage (33.8 kb) and an incomplete prophage (20.9 kb) are shown in gold and red, respectively. Bacterial genes are shown in orange and % GC in black. Phage genes are shown at each prophage region along the tracks. Prophages were identified using PHASTEST with FE9508BRB shown as the representative isolate.

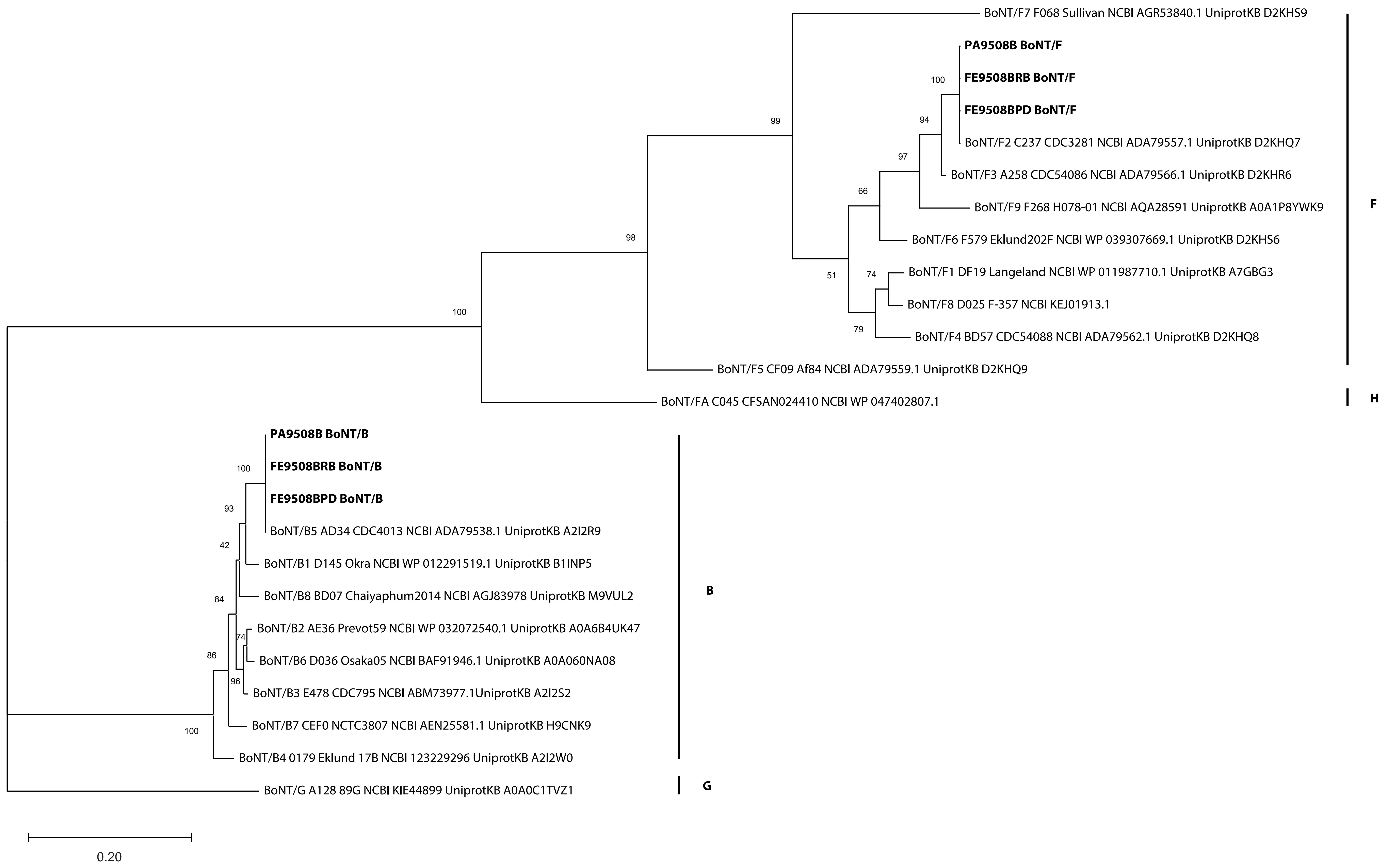
BoNT/F7 F068 Sullivan NCBI AGR53840.1 UniprotKB D2KHS9
PA9508B BoNT/F
FE9508BRB BoNT/F
FE9508BPD BoNT/F
BoNT/F2 C237 CDC3281 NCBI ADA79557.1 UniprotKB D2KHQ7
BoNT/F3 A258 CDC54086 NCBI ADA79566.1 UniprotKB D2KHR6
BoNT/F9 F268 H078-01 NCBI AQA28591 UniprotKB A0A1P8YWK9
BoNT/F6 F579 Eklund202F NCBI WP 039307669.1 UniprotKB D2KHS6
BoNT/F1 DF19 Langeland NCBI WP 011987710.1 UniprotKB A7GBG3
BoNT/F8 D025 F-357 NCBI KEJ01913.1
BoNT/F4 BD57 CDC54088 NCBI ADA79562.1 UniprotKB D2KHQ8
BoNT/F5 CF09 Af84 NCBI ADA79559.1 UniprotKB D2KHQ9
BoNT/FA C045 CFSAN024410 NCBI WP 047402807.1
F
H
PA9508B BoNT/B
FE9508BRB BoNT/B
FE9508BPD BoNT/B
BoNT/B5 AD34 CDC4013 NCBI ADA79538.1 UniprotKB A2I2R9
BoNT/B1 D145 Okra NCBI WP 012291519.1 UniprotKB B1INP5
BoNT/B8 BD07 Chaiyaphum2014 NCBI AGJ83978 UniprotKB M9VUL2
BoNT/B2 AE36 Prevot59 NCBI WP 032072540.1 UniprotKB A0A6B4UK47
BoNT/B6 D036 Osaka05 NCBI BAF91946.1 UniprotKB A0A060NA08
BoNT/B3 E478 CDC795 NCBI ABM73977.1UniprotKB A2I2S2
BoNT/B7 CEF0 NCTC3807 NCBI AEN25581.1 UniprotKB H9CNK9
BoNT/B4 0179 Eklund 17B NCBI 123229296 UniprotKB A2I2W0
BoNT/G A128 89G NCBI KIE44899 UniprotKB A0A0C1TVZ1
B
G
100
99
98
100
94
97
66
51
74
79
100
100
93
42
84
74
86
96
100
0.20

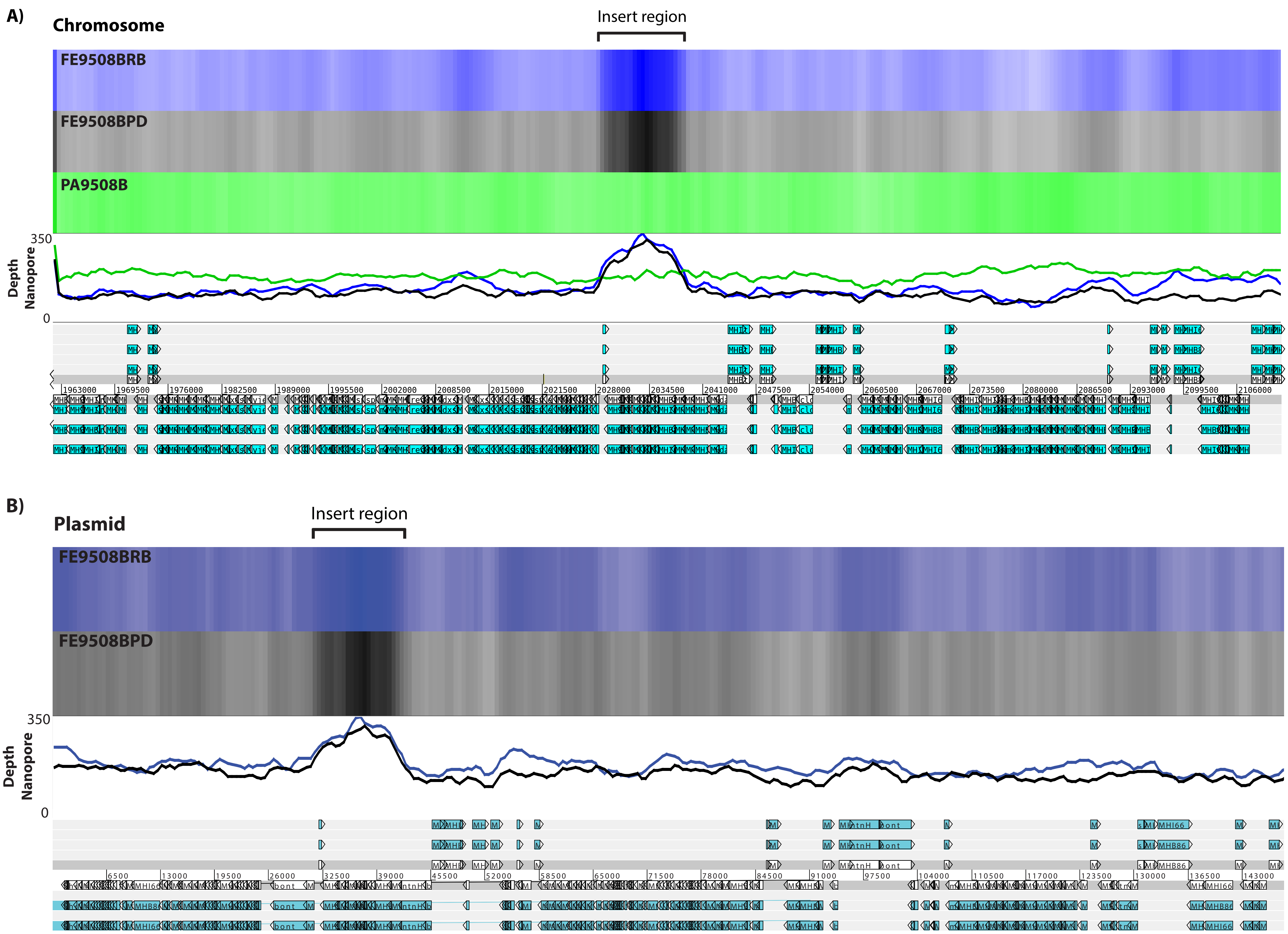
A)
Chromosome
Insert region
FE9508BRB
FE9508BPD
PA9508B
350
0
Depth Nanopore
1963000
1969500
1976000
1982500
1989000
1995500
2002000
2008500
2015000
2021500
2028000
2034500
2041000
2047500
2054000
2060500
2067000
2073500
2080000
2086500
2093000
2099500
2106000
B)
Plasmid
Insert region
FE9508BRB
FE9508BPD
350
0
Depth Nanopore
6500
13000
19500
26000
32500
39000
45500
52000
58500
65000
71500
78000
84500
91000
97500
104000
110500
117000
123500
130000
136500
143000

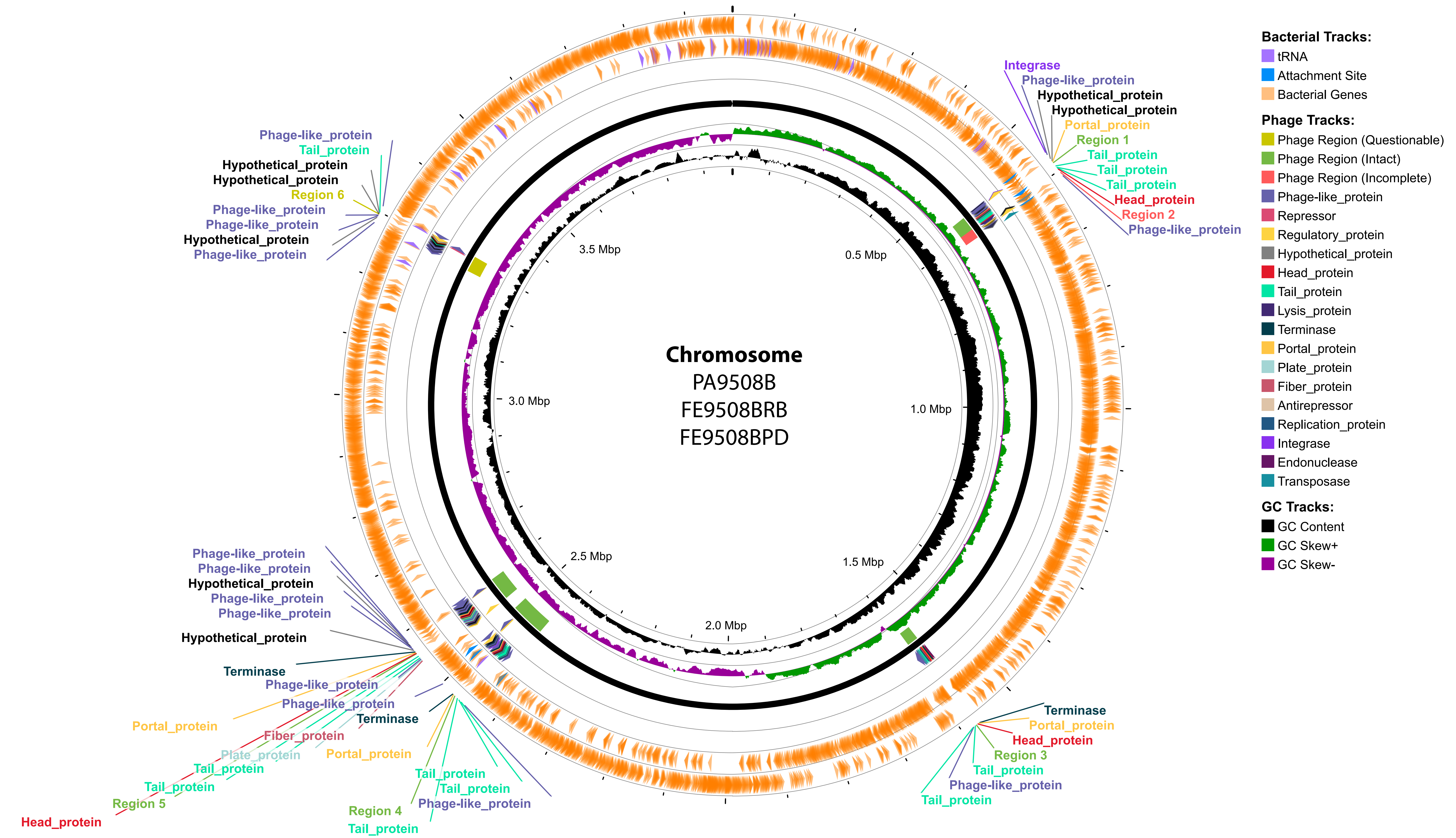

Chromosome
PA9508B
FE9508BRB
FE9508BPD
0.5 Mbp
1.0 Mbp
1.5 Mbp
2.0 Mbp
2.5 Mbp
3.0 Mbp
3.5 Mbp
Integrase
Phage-like_protein
Hypothetical_protein
Hypothetical_protein
Portal_protein
Region 1
Tail_protein
Tail_protein
Tail_protein
Head_protein
Region 2
Phage-like_protein
Terminase
Portal_protein
Head_protein
Region 3
Tail_protein
Phage-like_protein
Tail_protein
Terminase
Portal_protein
Tail_protein
Tail_protein
Phage-like_protein
Region 4
Tail_protein
Phage-like_protein
Phage-like_protein
Hypothetical_protein
Phage-like_protein
Phage-like_protein
Hypothetical_protein
Terminase
Phage-like_protein
Phage-like_protein
Portal_protein
Fiber_protein
Plate_protein
Tail_protein
Tail_protein
Region 5
Head_protein
Phage-like_protein
Tail_protein
Hypothetical_protein
Hypothetical_protein
Region 6
Phage-like_protein
Phage-like_protein
Hypothetical_protein
Phage-like_protein
Bacterial Tracks:
tRNA
Attachment Site
Bacterial Genes
Phage Tracks:
Phage Region (Questionable)
Phage Region (Intact)
Phage Region (Incomplete)
Phage-like_protein
Repressor
Regulatory_protein
Hypothetical_protein
Head_protein
Tail_protein
Lysis_protein
Terminase
Portal_protein
Plate_protein
Fiber_protein
Antirepressor
Replication_protein
Integrase
Endonuclease
Transposase
GC Tracks:
GC Content
GC Skew+
GC Skew-
